# The Neutrophil/Lymphocyte Ratio and Outcomes in Hospitalized Patients with Community-Acquired Pneumonia: A Retrospective Cohort Study

**DOI:** 10.3390/biomedicines12020260

**Published:** 2024-01-24

**Authors:** Aysun Tekin, Felix W. Wireko, Ognjen Gajic, Yewande E. Odeyemi

**Affiliations:** 1 Division of Nephrology and Hypertension, Department of Internal Medicine, Mayo Clinic, Rochester, MN 55905, USA; 2 Division of Pulmonary and Critical Care Medicine, Mayo Clinic, Rochester, MN 55905, USA; wireko.felix@mayo.edu (F.W.W.); gajic.ognjen@mayo.edu (O.G.); odeyemi.yewande@mayo.edu (Y.E.O.)

**Keywords:** community-acquired pneumonia, CAP, neutrophil/lymphocyte ratio, NLR, mortality, prognostication

## Abstract

We aimed to assess the prognostic role of the neutrophil/lymphocyte ratio (NLR) in community-acquired pneumonia (CAP) via a single-center retrospective cohort of hospitalized adult patients from 1/2009 to 12/2019. Patients were dichotomized into lower NLR (≤12) and higher NLR (>12). The primary outcome was mortality. ICU admission and hospital- and ICU-free days were secondary outcomes. The pneumonia severity index (PSI) and the NLR’s ability to predict outcomes was also tested. An NLR ≤12 was observed in 2513 (62.2%) patients and >12 in 1526 (37.8%). After adjusting for PSI, the NLR was not associated with hospital mortality (odds ratio [OR] 1.115; 95% confidence interval [CI] 0.774, 1.606; *p* = 0.559), but it was associated with a higher risk of ICU admission (OR 1.405; 95% CI 1.216, 1.624; *p* < 0.001). The PSI demonstrated acceptable discrimination for mortality (area under the receiver operating characteristic curve [AUC] 0.78; 95% CI 0.75, 0.82) which was not improved by adding the NLR (AUC 0.78; 95% CI 0.75, 0.82, *p* = 0.4476). The PSI’s performance in predicting ICU admission was also acceptable (AUC 0.75; 95% CI 0.74, 0.77) and improved by including the NLR (AUC 0.76, 95% CI 0.74, 0.77, *p* = 0.008), although with limited clinical significance. The NLR was not superior to the PSI for predicting mortality in hospitalized CAP patients.

## 1. Introduction

Community-acquired pneumonia (CAP) is an acute infection of the lung parenchyma and one of the leading causes of hospitalization and mortality in the United States [1], with more than 1.5 million annual hospitalizations and about 100,000 deaths [2]. Risk stratification and prognostication provide useful information on the disease trajectory and guide management [3]. Although several prognostic tools for CAP have been evaluated, the pneumonia severity index (PSI), Confusion, Uremia, Respiratory rate, Blood pressure, being 65 years of age and older (CURB-65), and the ATS/IDSA are the most validated and remain commonly used in CAP, with satisfactory outcomes [4,5,6]. Evaluations of the prognostic value of biomarkers like NT-pro-BNP [7], C-reactive protein, and procalcitonin have been less satisfactory when used individually [8]. The neutrophil/lymphocyte ratio (NLR) (the ratio of absolute neutrophil count to absolute lymphocyte count), is an easily measurable index that is receiving growing interest as a useful prognostic biomarker in several stressful conditions including infections. In CAP, multiple studies have shown conflicting results on the prognostic value of the NLR alone or in addition to clinical severity scores [7,9,10,11,12]. The addition of the NLR to the PSI and CURB-65 in one study did not improve the prediction of mortality [12]. More recently, a systematic review demonstrated a comparable prognostic value of the NLR to the PSI, CURB-65, CRP, procalcitonin, neutrophil count, lymphocyte count, and white blood cell count [13]. However, only a few studies in the systematic review confirmed an association between the NLR and adverse outcomes utilizing a multivariate analysis, thus resulting in limited consideration of confounding factors on mortality. Also, most studies focused on mortality only as an outcome, with minimal evaluation of other clinical outcomes including need for invasive and non-invasive mechanical support and admission to the intensive care unit (ICU). Further, most studies were limited by sample size.

The aim of this study was to evaluate the prognostic value of the NLR in predicting the clinical outcomes of patients who are hospitalized with CAP.

## 2. Materials and Methods

The institutional review board (IRB) at the Mayo Clinic approved this research as low-risk, denoted by the IRB number 17-011140, with a waiver of the requirement for written informed consent.

### 2.1. Design, Setting, and Participants

This study had a retrospective cohort design. All adult patients admitted to the Mayo Clinic in Rochester, Minnesota, between January 2009 and December 2019 who had community-acquired pneumonia (CAP) underwent screening. The presence of CAP was determined using International Classification of Diseases 9 (481–486) and 10 (J13, J15, and J18) codes, coupled with note searches. Community-acquired pneumonia was defined as an acute infection of the lung parenchyma exhibiting clinical symptoms (cough, fever, pleuritic chest pain, and dyspnea) and a new radiographic infiltrate not being acquired in the hospital or healthcare setting [14].

**Figure 1:**
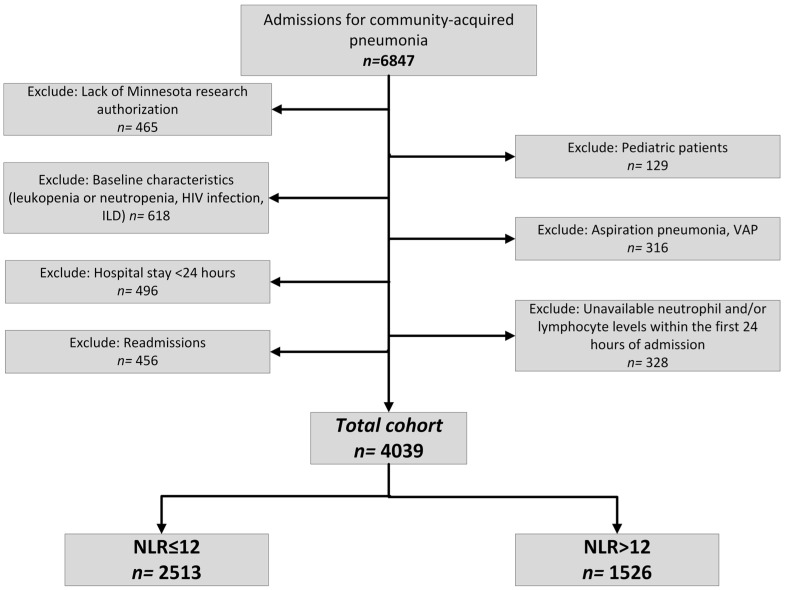
Flowchart of the identification of the patients in neutrophil/lymphocyte ratio analyses. HIV: human immunodeficiency virus, ILD: interstitial lung disease, NLR: neutrophil/lymphocyte ratio, VAP: ventilator-associated pneumonia.

The exclusion criteria were:

- The absence of Minnesota research authorization;
- Baseline conditions of human immune deficiency virus infection, interstitial lung disease, leukopenia, or neutropenia;
- Diagnoses of hospital-acquired pneumonia, ventilator-associated pneumonia, or aspiration pneumonia;
- Readmissions (only the earliest admission during the study period was included per individual);
- Hospital stays under 24 h;
- The absence of neutrophil or lymphocyte levels within the initial 24 h of admission (Figure 1).

### 2.2. Variable Definitions

The data were extracted by the Anesthesia Clinical Research Unit team using standardized and validated queries [15,16,17]. Extracted data included relevant demographic information, comorbidities (recorded as the presence of individual comorbidities alongside the Charlson Comorbidity Index [CCI]) [18], and disease severity indexes, evaluated via clinical scores. The medication exposure evaluation was limited to corticosteroids and vasopressors.

The white blood cell count, which measures the total number of leukocytes, is recorded as 10^9^/L in our institution, with the institutional normal falling between 3.4 and 9.6 × 10^9^/L. As per the differential components, the normal ranges for neutrophil and lymphocyte counts are considered to be 1.56 to 6.45 × 10^9^/L and 0.95 to 3.07 × 10^9^/L, respectively. Neutrophil and lymphocyte levels tested during the first 24 h of admission (the first one available if more than one result was available during the timeline) were recorded as continuous variables and used to calculate the neutrophil/lymphocyte ratio (NLR). Based on the literature, an a priori NLR cut-off of 12 was chosen (patients were compared as those whose NLR level was ≤12 vs. those >12) [13].

### 2.3. Outcomes

Primary outcomes included mortality during hospitalization and within six months of admission. Secondary outcomes encompassed the need for invasive and non-invasive mechanical ventilation (IMV) (NIMV), intensive care unit (ICU) admission, and hospital- and ICU-free days, calculated as days alive spent outside of the hospital or the ICU, respectively, within 28 days of admission, resulting in 0 for patients who died during the stay or had a length of stay of ≥28 days [19]. In a similar fashion, IMV-free days were also calculated and evaluated. The prediction of in-hospital mortality and the requirement for ICU admission using PSI-only and PSI combined with NLR were also calculated.

### 2.4. Statistical Analysis

The median, interquartile range (IQR) for continuous data and the numbers and frequencies for categorical variables were used to perform and present descriptive summary statistics. The chi-square test was used to examine categorical data, while the Mann–Whitney U test was used to evaluate continuous variables. The NLR ≤ 12 group was used as the reference for comparing the two groups. Results were reported using odds ratios (ORs), *p* values, estimates, and 95% confidence intervals (CIs). The analyses were adjusted for the baseline conditions and severity of the patients, measured by the PSI score using binary logistic regression and linear regression, depending on the characteristics of the outcomes of interest. As PSI already includes baseline characteristics such as age, gender, and certain comorbid conditions, no additional covariates were deemed necessary for the multivariable calculations comparing the NLR groups.

To better understand NLR’s association with outcomes, the following sensitivity analyses were conducted:

- Evaluating the impact of increasing NLR as a continuous variable;
- Classifying patients into four categories based on quartiles (quartile [Q] #1 NLR < 5.13, Q #2 NLR ≥ 5.13 and <9.26, Q #3 NLR ≥ 9.26 and <16.35, Q #4 NLR ≥ 16.35; Q #1 was the reference group);
- Limiting the analyses to patients with neutrophilia (defined as having an absolute neutrophil count of >6.45 × 10^9^, the institutional cut-off level);
- Limiting the analyses to patients with lymphopenia (defined as having an absolute lymphocyte count of <0.95 × 10^9^, the institutional cut-off level).

Finally, C-statistics was used to examine the NLR and PSI’s predictive ability for mortality and ICU admission. This included creating a receiver operating characteristic (ROC) curve and determining the area under the curve (AUC), as well as the 95% CI The potential contribution of NLR to the PSI was also evaluated. The ROC curves for both approaches were compared using DeLong’s test [20]. A two-sided *p*-value of 0.05 was considered significant. IBM SPSS v27.0 (IBM Statistical Package for Social Sciences Statistics for Windows, Armonk, NY, USA) and MedCalc Statistical Software v19.1 (MedCalc Software bv, Ostend, Belgium) were used to perform the calculations.

## 3. Results

Following the application of exclusion criteria, 4039 patients out of the initial 6847 were included in the cohort (Figure 1).

### 3.1. Comparison of Patients with an NLR ≤ 12 vs. >12

The primary analysis compared 2513 patients with NLR ≤ 12 and 1526 with NLR > 12. Table 1 illustrates the distribution of baseline characteristics across both groups. Although there were some differences in the distribution of specific comorbidities, the baseline comorbidity burdens were generally well-balanced, as indicated by a comparable CCI (median [IQR] = 7 [5,10] and 7 [5,9] for NLR ≤ 12 and >12 groups, respectively, *p* = 0.541).

Table 2 outlines the distribution of the primary outcomes and the results of the multivariable analysis. The odds of ICU admission were higher in patients with NLR > 12 (*n* = 710, 46.5%) compared to those with NLR ≤ 12 (*n* = 869, 34.6%) (adjusted OR [95% CI] = 1.41 [1.22, 1.62]).

### 3.2. Sensitivity Analyses

#### 3.2.1. Evaluation of the Impact of the NLR as a Continuous Variable

Upon analyzing the relationship between an increasing NLR and various outcomes without categorization, we observed a rise in the odds of both hospital mortality and ICU admission as the NLR increased (adjusted OR [95% CI] = 1.01 [1.00, 1.02], *p* = 0.047 and adjusted OR [95% CI] = 1.02 [1.01, 1.02], *p* < 0.001 for hospital mortality and ICU admission, respectively). Additionally, higher NLR levels were associated with a significant decrease in the number of hospital-free days (adjusted estimate [95% CI] = −0.02 [−0.04, −0.01]). Although univariate analyses suggested a significant association between the NLR and mortality within six months after admission, as well as the need for mechanical ventilation, these associations did not remain significant after adjusting for the PSI (adjusted OR [95% CI] = 1.0 [1.0, 1.01], *p* = 0.693 and adjusted OR [95% CI] = 1.0 [1.0, 1.01], *p* = 0.198 for mortality at six months and the requirement for mechanical ventilation, respectively).

#### 3.2.2. Four-Group Comparison Based on NLR Quartiles

Supplementary Table S1 and Table 3 illustrate the distribution of baseline characteristics and outcomes among the four groups of patients based on NLR levels. Similar to the primary analyses, the need for ICU admission was significantly different in patients belonging to Q#3 (*n* = 405, 40.1%) and Q#4 (*n* = 492 (48.7%) compared to Q#1 (*n* = 319, 31.6%) (adjusted OR [95% CI] = 1.38 [1.16, 1.69] and 1.64 [1.34, 2.01] for Q#3 and Q#4, respectively).

#### 3.2.3. Analysis among Patients with Neutrophilia

Supplementary Tables S2 and S3 depict the distribution of baseline characteristics and outcomes for the comparative analysis among the 2989 patients with neutrophilia.

#### 3.2.4. Analysis among Patients with Lymphopenia

Supplementary Tables S4 and S5 showcase the distribution of baseline characteristics and outcomes for the comparative analysis among the 1905 patients with lymphopenia.

### 3.3. Discriminatory Performance

**Figure 2:**
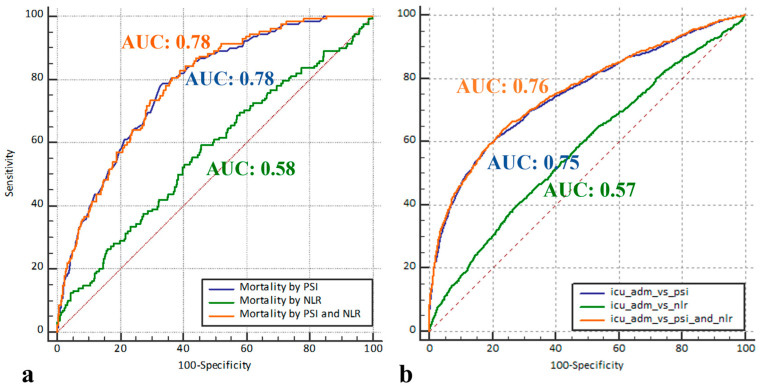
Receiver operating characteristic curves for pneumonia severity index (PSI) only, neutrophil/lymphocyte ratio (NLR) only, and PSI combined with NLR in detecting (**a**) in-hospital mortality (the area under the receiver operating characteristic curve (AUC) was 0.78 (95% CI, 0.75–0.82) for PSI only and PSI combined with NLR approaches, while it was 0.57 (95% CI, 0.52–0.62) for NLR only) and (**b**) the requirement for intensive care unit admission (the AUC was 0.75 (95% CI, 0.74–0.77), 0.58 (95% CI, 0.56–0.6), and 0.76 (95% CI: 0.74–0.77) for PSI only, NLR only, and PSI combined with NLR, respectively). ICU: intensive care unit.

Supplementary Table S6 presents the distribution of baseline characteristics between patients who died during their hospital stay and those who were discharged alive. The distinguishing capacity of the NLR for patients who died during hospitalization was poor (AUC [95% CI] = 0.57 [0.52, 0.62]). The discriminatory capacity of the PSI for patients who died during hospitalization was acceptable (AUC [95% CI] = 0.78 [0.75, 0.82]) and did not considerably improve with the addition of the NLR (AUC [95% CI] = 0.78 [0.75, 0.82]) (Figure 2a), with a negligible difference between the AUC for PSI versus PSI and NLR of 0.0025 (95% CI: −0.0039, 0.0089, *p* = 448).

The discriminatory capacity of the NLR for patients requiring an ICU admission was poor (AUC [95% CI] = 0.58 [0.56, 0.6]). The distinguishing capacity of the PSI for patients requiring an ICU stay was acceptable (AUC [95% CI] = 0.75 [0.74, 0.77]) and significantly improved with the addition of the NLR (AUC [95% CI] = 0.76 [0.74, 0.77]) (Figure 2b), with a difference between the AUC for PSI versus PSI and NLR of 0.0041 (95% CI: 0.001, 0.007, *p* = 0.008).

## 4. Discussion

In this large single-center retrospective cohort of hospitalized patients with community-acquired pneumonia, we found that a higher NLR (>12) was not associated with an increased odds for in-hospital mortality and 6-month mortality. Notably, there was a significant difference in the severity of illness as measured by the PSI, CURB-65, and APACHE III scores between the subgroups (NLR ≤ 12 and >12), and a significant association with mortality following univariate analysis. However, after adjusting for baseline comorbid conditions and the severity of illness using the PSI, the association between a higher NLR and mortality became insignificant. Additional adjusted analysis with the NLR as a continuous variable revealed a barely significant association between the NLR and in-hospital mortality. On assessment of secondary outcomes, a higher NLR was only found to be associated with an increased odds of ICU admission in this cohort, while no association was observed with other secondary outcomes including the need for invasive and non-invasive mechanical ventilation. Further adjusted analysis with the NLR as a continuous variable also revealed a significant association with ICU admission and hospital-free days, but no association with other secondary outcomes. These findings were consistent on additional sensitivity analysis evaluating outcomes in NLR quartiles, with an increasing odds of ICU admission in the third and fourth quartiles. Interestingly, the increased odds of ICU admission were only observed in the subset of patients with a high NLR and co-existing lymphopenia compared to patients with concomitant neutrophilia. Additionally, an increased odds for a need of both invasive and non-invasive mechanical ventilation were noted in this lymphopenic subgroup. Potential explanations include the presence of other confounding factors like the increasing use of non-invasive mechanical support for high-risk extubation patients such as COPD. Finally, in this cohort, the discriminatory capacity of the NLR in predicting both mortality and ICU admission was inferior to the PSI. Moreover, the addition of the NLR to the PSI did not improve the prediction of the mortality model. The improvement with the addition of the NLR to the PSI for predicting ICU admission, albeit statistically significant, was very small and not clinically meaningful.

The utility of the NLR in predicting adverse outcomes in CAP has been previously reported in other studies [7,8,10,11,21,22]. However, these studies reported conflicting results and were limited by sample size. Further, only a few studies confirmed an association between the NLR and mortality by multivariate analysis. In a recent systematic review of nine studies (*n* = 3340), the association between a higher NLR and mortality was observed to be significant. An NLR cut-off value >10 in this systematic review was found to predict mortality compared to the PSI, CURB-65, and other biomarkers including C-reactive protein, lymphocytes, neutrophils, and WBC [13]. A higher sensitivity and specificity were observed at a cut-off value between 11.2 and 13.4. In contrast, our results demonstrated a barely significant association between an elevated NLR and mortality, and also a poor discriminatory ability of the NLR in predicting mortality compared to the PSI. Although the results of a retrospective study by Postma et al., the largest study included in the systematic review (*n* = 1549), reported a significant association between the NLR and mortality based on bivariate analysis, this was absent in the subsequent multivariate analysis. In addition, the lack of improvement in the PSI model with the addition of the NLR, as seen in our study, was similarly observed in the large study by Postma et al. [12]. Therefore, our result in this larger cohort further limits the clinical applicability of utilizing the NLR as a prognostic tool for mortality, alone or in combination with severity scores, in CAP. Further studies, including prospective studies and an updated systematic review will be needed.

In comparison with other prior studies evaluating the prognostic value of the NLR in CAP, our study further found a significant association between the NLR and need for ICU admission, an important outcome that is rarely assessed. In addition, other outcomes, including the need for mechanical ventilation, hospital-free days, and ventilator-free days, were all assessed. Moreover, our study explored the outcomes in subset of patients with neutrophilia versus lymphopenia, with significant associations observed in the lymphopenia group.

The strengths of our study compared to prior similar studies include the large sample size (the largest sample size to our knowledge), the multivariable analysis adjusting for the severity of illness and comorbidities, the exploration of other clinical outcomes in addition to mortality, and the sensitivity analysis evaluating specific subgroups. As this was a single-center cohort study, findings reported are reflective of characteristics observed in a large academic center. Other limitations include the potential bias existing within the dataset, missing data, the lack of microbiological data, and other unmeasured confounders.

## 5. Conclusions

In this study, the neutrophil/lymphocyte ratio was not superior to the pneumonia severity index for predicting in-hospital mortality in patients who were hospitalized with community-acquired pneumonia. This limits its applicability as a prognostic enrichment tool, but additional studies are needed for assessing its use as a predictive enrichment tool.

## Figures and Tables

**Table 1:** Demographics and clinical characteristics of patients.

Variables	Total (*n* = 4039)	NLR ≤ 12 (*n* = 2513)	NLR > 12 (*n* = 1526)	*p*, OR (95% CI)
Age, median (IQR)	78 (65, 88)	77 (65, 88)	80 (67, 88)	0.003
Sex, Male, no. (%)	2172 (53.8%)	1293 (51.5%)	879 (57.6%)	<0.001, 1.28 (1.13, 1.46)
Race, no. (%)				0.038
White	3805 (94.2%)	2349 (93.5%)	1456 (95.4%)	
Asian	42 (1.0%)	31 (1.2%)	11 (0.7%)	
African American	64 (1.6%)	50 (2.0%)	14 (0.9%)	
Others	128 (3.2%)	83 (3.3%)	45 (2.9%)	
Ethnicity, no. (%)				0.401
Non-Hispanic	3897 (96.5%)	2423 (96.4%)	1474 (96.6%)	
Hispanic	65 (1.6%)	45 (1.8%)	20 (1.3%)	
Unknown/Other	77 (1.9%)	45 (1.8%)	32 (2.1%)	
Comorbidities, no. (%)				
Congestive heart failure	1118 (27.7%)	703 (28.0%)	415 (27.2%)	0.592, 0.96 (0.83, 1.11)
COPD	1310 (32.4%)	740 (29.4%)	570 (37.4%)	<0.001, 1.43 (1.25, 1.63)
Diabetes	1220 (30.2%)	790 (31.4%)	430 (28.2%)	0.029, 0.86 (0.74, 0.98)
Chronic kidney disease	1159 (28.7%)	724 (28.8%)	435 (28.5%)	0.836, 0.99 (0.86, 1.13)
Malignancy	938 (23.5%)	576 (22.9%)	372 (24.4%)	0.290, 1.08 (0.93, 1.26)
Neutrophil/lymphocyte ratio, median, (IQR)	9.26 (5.13, 16.35)	6.09 (3.80, 8.67)	19.71 (14.86, 27.88)	
Clinical Severity Scores				
Pneumonia Severity Index, median (IQR)	113 (87, 141)	109 (83, 136)	119 (93, 148)	<0.001
CURB 65	3 (2, 3)	3 (2, 3)	3 (2, 4)	<0.001
APACHE III	65 (52, 80)	64 (51, 79)	66 (53, 81)	0.013
Need for vasopressor during hospitalization, no. (%)	888 (22.0%)	512 (20.4%)	376 (24.6%)	0.002, 1.28 (1.10, 1.49)

APACHE: Acute Physiology and Chronic Health Evaluation, CI: confidence interval, COPD: Chronic Obstructive Pulmonary Disease, NLR: neutrophil/lymphocyte ratio, IQR: interquartile range, OR: odds ratio.

**Table 2:** Primary and secondary outcomes based on NLR levels.

Variables	Total (*n* = 4039)	NLR ≤ 12 (*n* = 2513)	NLR > 12 (*n* = 1526)	Univariate Analysis		Multivariate Analysis *	
				Odds Ratio (95% CI)	*p* Value	Odds Ratio (95% CI)	*p* Value
Hospital mortality, no. (%)	128 (3.2%)	69 (2.7%)	59 (3.9%)	1.43 (1.00, 2.03)	0.049	1.12 (0.77, 1.61)	0.559
Mortality at 6 months, no. (%)	638 (15.8%)	375 (14.9%)	263 (%17.2)	1.19 (1.00, 1.41)	0.051	0.99 (0.83, 1.19)	0.923
Need for invasive mechanical ventilation, no. (%)	704 (17.4%)	406 (16.2%)	298 (19.5%)	1.26 (1.07, 1.49)	0.006	1.08 (0.91,1.28)	0.395
Need for invasive and non-invasive mechanical ventilation, no. (%)	1348 (33.4%)	789 (31.4%)	559 (36.6%)	1.26 (1.11, 1.44)	<0.001	1.08 (0.93,1.24)	0.312
ICU admission	1579 (39.1%)	869 (34.6%)	710 (46.5%)	1.65 (1.45, 1.87)	<0.001	1.41 (1.22, 1.62)	<0.001
					*p* value	Estimate (95% CI)	*p* value
Hospital-free days, median (IQR)	24.01 (21.04, 25.44)	24.13 (21.38, 25.65)	23.78 (20.23, 25.29)		<0.001	−0.34 (−0.72, 0.04)	0.081
Invasive ventilator-free days, median (IQR)	26.29 (23.67, 27.27)	26.19 (23.19, 27.28)	26.40 (23.97, 27.19)		0.510	0.96 (−0.31, 2.23)	0.139

CI: confidence interval, ICU: intensive care unit, IQR: interquartile range, NLR: neutrophil/lymphocyte ratio. * Data were analyzed using multivariable regression models adjusting for pneumonia severity index. NLR ≤ 12 was the reference.

**Table 3:** Primary and secondary outcomes of patients according to their neutrophil/lymphocyte ratio levels *.

Variables	Quartile #1 (*n* = 1010)	Quartile #2 (*n* = 1010)	Quartile #3 (*n* = 1009)	Quartile #4 (*n* = 1010)	Univariable Analysis	Multivariable Analysis **		
					*p* Value		Odds Ratio (95% CI)	*p* Value
Hospital mortality, no. (%)	24 (2.4)	27 (2.7)	34 (3.4)	43 (4.3)	0.075	Q#2	1.09 (0.62, 1.93)	0.763
						Q#3	1.3 (0.76, 2.23)	0.343
						Q#4	1.28 (0.76, 2.16)	0.352
Mortality at 6 months, no. (%)	162 (16)	144 (14.3)	152 (15.1)	180 (17.8)	0.147	Q#2	0.83 (0.64, 1.07)	0.149
						Q#3	0.84 (0.66, 1.08)	0.185
						Q#4	0.86 (0.68, 1.1)	0.238
Need for invasive mechanical ventilation, no. (%)	152 (15)	165 (16.3)	185 (18.3)	202 (20)	0.018	Q#2	1.07 (0.84, 1.37)	0.581
						Q#3	1.19 (0.93, 1.51)	0.164
						Q#4	1.12 (0.88, 1.43)	0.341
Need for invasive and non-invasive mechanical ventilation, no. (%)	300 (29.7)	324 (32.1)	346 (34.3)	378 (37.4)	0.002	Q#2	1.08 (0.89, 1.32)	0.422
						Q#3	1.15 (0.95, 1.4)	0.158
						Q#4	1.12 (0.92, 1.37)	0.254
ICU admission	319 (31.6)	363 (35.9)	405 (40.1)	492 (48.7)	<0.001	Q#2	1.19 (0.97, 1.46)	0.090
						Q#3	1.38 (1.13, 1.69)	0.002
						Q#4	1.64 (1.34, 2.01)	<0.001
					*p* value		Estimate (95% CI)	*p* value
Hospital-free days, median (IQR)	24.3 (22.06, 25.88)	24.01 (21.12, 25.48)	23.98 (21, 25.41)	23.55 (19.96, 25.23)	<0.001	Q#2	−0.41 (−0.93, 0.11)	0.120
						Q#3	−0.43 (−0.95, 0.09)	0.104
						Q#4	−0.63 (−1.15, −0.11)	0.017

CI: confidence interval, ICU: intensive care unit, IQR: interquartile range, Q: quartile. * Neutrophil-lymphocyte ratio levels per quartiles: Quartile #1, <5.13; Quartile #2, ≥5.13 and <9.26; Quartile #3, ≥9.26 and <16.35; Quartile #4, ≥16.35. ** Data were analyzed using multivariable regression models adjusting for pneumonia severity index. Quartile #1 was the reference.

## Data Availability

The data that support the findings of this study are available from the corresponding author, A.T., upon reasonable request.

## Supplementary Materials

The following supporting information can be downloaded at https://www.mdpi.com/article/10.3390/biomedicines12020260/s1: Table S1: Demographics and clinical characteristics of patients according to their neutrophil/lymphocyte ratio levels; Table S2: Demographics and clinical characteristics of patients who had neutrophilia; Table S3: Primary and secondary outcomes of patients who had neutrophilia based on NLR; Table S4: Demographics and clinical characteristics of patients who had lymphopenia; Table S5: Primary and secondary outcomes of patients who had lymphopenia based on NLR; Table S6: Demographics and clinical characteristics of patients according to their in-hospital mortality status.
