# Supplementary material for: The Neutrophil/Lymphocyte Ratio and Outcomes in Hospitalized Patients with Community-Acquired Pneumonia: A Retrospective Cohort Study
Source: Biomedicines. 2024 Jan 24;12(2):260. doi: 10.3390/biomedicines12020260 (PMC10886607; doi:10.3390/biomedicines12020260)
Supplement: Supplementary file 1 [file biomedicines-12-00260-s001.zip › biomedicines-2827485-supplementary.pdf]

Supplementary Table S1. Demographics and clinical characteristics of patients according to their neutrophil-lymphocyte ratio levels[*]:

Variables	Quartile #1 (*n*=1010)	Quartile #2 (*n*=1010)	Quartile #3 (*n*=1009)	Quartile #4 (*n*=1010)	*P* value
**Age, median (IQR)**	76 (63, 88)	78 (65, 88)	79 (67, 88)	79 (67, 88)	**0.020**
**Sex , no. (%)**					**<0.001**
Male	499 (49.4)	519 (51.4)	563 (55.8)	591 (58.5)	
**Race, no. (%)**					**0.002**[**]
White	928 (91.9)	955 (94.6)	954 (94.5)	968 (95.8)	
Asian	15 (1.5)	12 (1.2)	9 (0.9)	6 (0.6)	
African American	32 (3.2)	14 (1.4)	11 (1.1)	7 (0.7)	
Others	35 (3.5)	29 (2.8)	35 (3.5)	29 (2.9)	
**Ethnicity, no. (%)**					0.650
Non-Hispanic	978 (96.8)	972 (96.2)	970 (96.1)	977 (96.7)	
Hispanic	16 (1.6)	21 (2.1)	15 (1.5)	13 (1.3)	
Unknown/Other	16 (1.6)	17 (1.7)	24 (2.4)	20 (2)	
**Comorbidities, no. (%)**					
Congestive heart failure	297 (29.4)	287 (28.4)	256 (25.4)	278 (27.5)	0.215
COPD	286 (28.3)	308 (30.5)	309 (30.6)	407 (40.3)	**<0.001**
Diabetes	335 (33.2)	305 (30.2)	304 (30.1)	276 (27.3)	**0.042**
Chronic kidney disease	299 (29.6)	296 (29.3)	258 (25.6)	306 (30.3)	0.083
Malignancy	226 (22.4)	249 (24.7)	214 (21.2)	259 (25.6)	0.072
**Neutrophil/lymphocyte ratio, median, (IQR)**	3.35 (2.37, 4.27)	7.04 (6.09, 8.19)	12.06 (10.65, 14.05)	24.55 (19.79, 33.82)	**0.000**
**Clinical Severity Scores**					
Pneumonia Severity Index, median (IQR)	105 (80, 136)	111 (85, 137)	113 (88, 139)	121 (95, 152)	**<0.001**
CURB 65	2 (2, 3)	3 (2, 3)	3 (2, 3)	3 (2, 4)	**<0.001**
APACHE III	64 (51, 80)	65 (51, 78)	62 (49, 77)	68 (56, 82)	**<0.001**

APACHE: Acute Physiology and Chronic Health Evaluation, CI: Confidence interval, COPD: Chronic Obstructive Pulmonary Disease, NLR: Neutrophil Lymphocyte ratio, IQR: interquartile range, OR: Odds ratio.

[*] neutrophil-lymphocyte ratio levels per quartiles: Quartile #1, <5.13; Quartile #2, ≥5.13 and <9.26; Quartile #3, ≥9.26 and <16.35; Quartile #4, ≥16.35.

[**] To address the issue of a high number of cells having an expected count of less than 5, the race categories were merged for comparison purposes into two groups: White and others.

<u>Supplementary Table S2.</u> Demographics and clinical characteristics of patients who had neutrophilia*:

Variables	Total (*n*=2989)	NLR ≤ 12 (*n*=1562)	NLR > 12 (*n*=1427)	*p*, OR (95% C.I.)
**Age, median (IQR)**	78 (66, 88)	76 (65, 87)	80 (67, 89)	**<0.001**
**Sex , no. (%)**				
Male	1607 (53.8)	795 (50.9)	812 (56.9)	**0.001, 1.27 (1.10, 1.47)**
**Race, no. (%)**				**0.353****
White	2837 (94.9)	1474 (94.4)	1363 (95.5)	
Asian	26 (0.9)	15 (1)	11 (0.8)	
African American	39 (1.3)	26 (1.7)	13 (0.9)	
Others	87 (2.9)	47 (3)	40 (2.8)	
**Ethnicity, no. (%)**				0.366
Non-Hispanic	2888 (96.6)	1508 (96.5)	1380 (96.7)	
Hispanic	44 (1.5)	27 (1.7)	17 (1.2)	
Unknown/Other	57 (1.9)	27 (1.7)	30 (2.1)	
**Comorbidities, no. (%)**				
Congestive heart failure	807 (27)	416 (26.6)	391 (27.4)	0.637, 1.04 (0.89, 1.22)
COPD	1020 (34.1)	484 (31)	536 (37.6)	**<0.001, 1.34 (1.15, 1.56)**
Diabetes	897 (30)	488 (31.2)	409 (28.7)	0.124, 0.88 (0.76, 1.03)
Chronic kidney disease	832 (27.8)	427 (27.3)	405 (28.4)	0.525, 1.05 (0.90, 1.24)
Malignancy	681 (22.8)	339 (21.7)	342 (24)	0.141, 1.14 (0.96, 1.35)
**Neutrophil/lymphocyte ratio, median, (IQR)**	11.55 (7.16, 19.55)	7.35 (5.16, 9.57)	19.99 (15.01, 28.42)	**<0.001**
**Clinical Severity Scores**				
Pneumonia Severity Index, median (IQR)	113 (88, 142)	109 (84, 136)	119 (92, 147)	**<0.001**
CURB 65	3 (2, 3)	3 (2, 3)	3 (2, 4)	**<0.001**
APACHE III	65 (52, 79)	63 (49, 79)	66 (53, 81)	**0.010**

APACHE: Acute Physiology and Chronic Health Evaluation, CI: Confidence interval, COPD: Chronic Obstructive Pulmonary Disease, NLR: Neutrophil Lymphocyte ratio, IQR: interquartile range, OR: Odds ratio.

* Neutrophilia was defined as having an absolute neutrophile count of > $6.45*10^9$.

** To address the issue of a high number of cells having an expected count of less than 5, the race categories were merged for comparison purposes into two groups: White and others.

Supplementary Table S3. **Primary and Secondary Outcomes of patients who had neutrophilia* based on NLR:**

**Variables**	**Total (*n*=2989)**	**NLR ≤ 12 (*n*=1562)**	**NLR > 12 (*n*=1427)**	**Univariable analysis**		**Multivariable analysis****	
				**Odds ratio (95% CI)**	***P* value**	**Odds ratio (95% CI)**	***P* value**
**Hospital mortality, no. (%)**	89 (3)	40 (2.6)	49 (3.4)	1.35 (0.89, 2.07)	0.161	1.07 (0.69, 1.66)	0.758
**Mortality at 6 months, no. (%)**	438 (14.7)	200 (12.8)	238 (16.7)	1.36 (1.11, 1.67)	0.003	1.17 (0.95, 1.45)	0.141
**Need for invasive mechanical ventilation, no. (%)**	545 (18.2)	269 (17.2)	276 (19.3)	1.15 (0.96, 1.39)	0.134	0.99 (0.81, 1.2)	0.885
**Need for invasive and non-invasive mechanical ventilation, no. (%)**	1044 (34.9)	524 (33.5)	520 (36.4)	1.14 (0.98, 1.32)	0.097	0.96 (0.82, 1.13)	0.610
**ICU admission**	1240 (41.5)	586 (37.5)	654 (45.8)	1.41 (1.22, 1.63)	<0.001	1.17 (0.99, 1.38)	0.062
					***P* value**	**Estimate (95% CI)**	***P* value**
**Hospital free days,median (IQR)**	23.99 (21.01, 25.4)	24.1 (21.34, 25.61)	23.85 (20.34, 25.31)		<0.001	-0.25 (-0.67, 0.17)	0.249

CI: Confidence interval, ICU: intensive care unit, IQR: interquartile range, NLR: Neutrophil Lymphocyte ratio, OR: Odds ratio.

* Neutrophilia was defined as having an absolute neutrophile count of > $6.45*10^9$.
**Data were analyzed using multivariable regression models adjusting for pneumonia severity index. NLR ≤ 12 was the reference.

<u>Supplementary Table S4.</u> Demographics and clinical characteristics of patients who had lymphopenia*:

Variables	Total (*n*=1905)	NLR ≤ 12 (*n*=629)	NLR > 12 (*n*=1276)	*p*, OR (95% C.I.)
**Age, median (IQR)**	80 (68, 89)	80 (68, 89)	80 (68, 88)	0.651
**Sex , no. (%)**				0.300, 1.11 (0.91, 1.34)
Male	1122 (58.9)	360 (57.2)	762 (59.7)	
**Race, no. (%)**				0.060, 0.68 (0.45, 1.02)**
White	1804 (94.7)	587 (93.3)	1217 (95.4)	
Asian	18 (0.9)	8 (1.3)	10 (0.8)	
African American	17 (0.9)	5 (0.8)	12 (0.9)	
Others	66 (3.5)	29 (4.6)	37 (2.9)	
**Ethnicity, no. (%)**				0.624
Non-Hispanic	1842 (96.7)	610 (97)	1232 (96.6)	
Hispanic	28 (1.5)	10 (1.6)	18 (1.4)	
Unknown/Other	35 (1.8)	9 (1.4)	26 (2)	
**Comorbidities, no. (%)**				
Congestive heart failure	530 (27.8)	175 (27.8)	355 (27.8)	1.000, 1.00 (0.81, 1.24)
COPD	673 (35.3)	186 (29.6)	487 (38.2)	**<0.001, 1.47 (1.20, 1.81)**
Diabetes	524 (27.5)	169 (26.9)	355 (27.8)	0.661, 1.05 (0.85, 1.30)
Chronic kidney disease	574 (30.1)	182 (28.9)	392 (30.7)	0.424, 1.09 (0.88, 1.34)
Malignancy	489 (25.7)	167 (26.6)	322 (25.2)	0.537, 0.93 (0.75, 1.16)
**Neutrophil/lymphocyte ratio, median, (IQR)**	15.88 (10.19, 25.01)	8.19 (6.09, 10.13)	21.25 (15.78, 30.13)	**<0.001**
**Clinical Severity Scores**				
Pneumonia Severity Index, median (IQR)	118 (93, 145)	115 (90, 139)	121 (94, 148)	**<0.001**
CURB 65	3 (2, 4)	3 (2, 3)	3 (2, 4)	**<0.001**
APACHE III	66 (53, 81)	63 (52, 79)	67 (54, 82)	0.102

APACHE: Acute Physiology and Chronic Health Evaluation, CI: Confidence interval, COPD: Chronic Obstructive Pulmonary Disease, NLR: Neutrophil Lymphocyte ratio, IQR: interquartile range, OR: Odds ratio.

* Lymphopenia was defined as having an absolute lymphocyte count of < $0.95*10^9$.

** To address the issue of a high number of cells having an expected count of less than 5, the race categories were merged for comparison purposes into two groups: White and others.

<u>Supplementary Table S5.</u> Primary and Secondary Outcomes of patients who had lymphopenia* based on NLR:

**Variables**	**Total (*n*=1905)**	**NLR ≤ 12 (*n*=629)**	**NLR > 12 (*n*=1276)**	**Univariable analysis**		**Multivariable analysis****	
				**Odds ratio (95% CI)**	***P* value**	**Odds ratio (95% CI)**	***P* value**
**Hospital mortality, no. (%)**	78 (4.1)	26 (4.1)	52 (4.1)	0.99 (0.61, 1.59)	0.952	0.84 (0.51, 1.38)	0.491
**Mortality at 6 months, no. (%)**	340 (17.8)	114 (18.1)	226 (17.7)	0.97 (0.76, 1.25)	0.825	0.86 (0.67, 1.12)	0.270
**Need for invasive mechanical ventilation, no. (%)**	341 (17.9)	96 (15.3)	245 (19.2)	1.32 (1.02, 1.71)	0.035	1.18 (0.9, 1.55)	0.226
**Need for invasive and non-invasive mechanical ventilation, no. (%)**	650 (34.1)	182 (28.9)	468 (36.7)	1.42 (1.16, 1.75)	<0.001	**1.3 (1.05, 1.62)**	**0.018**
**ICU admission**	812 (42.6)	218 (34.7)	594 (46.6)	1.64 (1.35, 2.00)	<0.001	**1.54 (1.23, 1.92)**	**0.000**
					***P* value**	**Estimate (95% CI)**	***P* value**
**Hospital free days,median (IQR)**	23.8 (20.3, 25.32)	23.82 (20.55, 25.42)	23.79 (20.21, 25.26)		0.234	0.08 (-0.52, 0.68)	0.800

CI: Confidence interval, ICU: intensive care unit, IQR: interquartile range, NLR: Neutrophil Lymphocyte ratio, OR: Odds ratio.

* Lymphopenia was defined as having an absolute lymphocyte count of < $0.95*10^9$.
**Data were analyzed using multivariable regression models adjusting for pneumonia severity index. NLR ≤ 12 was the reference.

Supplementary Table S6. Demographics and clinical characteristics of patients according to their in-hospital mortality status:

Variables	Total (*n*=4039)	Discharged alive (*n*=3911)	Died during admisison (*n*=128)	*p*, OR (95% C.I.)
**Age, median (IQR)**	78 (65, 88)	78 (65, 88)	79 (70, 91)	**0.035**
**Sex , no. (%)**				**0.011, 1.61 (1.11, 2.33)**
Male	2172 (53.8)	2089 (53.4)	83 (64.8)	
**Race, no. (%)**				0.542, 1.24 (0.62, 2.47)*
White	3805 (94.2)	3686 (94.2)	119 (93)	
Asian	42 (1)	42 (1.1)	0	
African American	64 (1.6)	63 (1.6)	1 (0.8)	
Others	128 (3.2)	120 (3.1)	8 (6.3)	
**Ethnicity, no. (%)**				**0.005, 2.71 (1.43, 5.16)****
Non-Hispanic	3897 (96.5)	3780 (96.7)	117 (91.4)	
Hispanic	65 (1.6)	64 (1.6)	1 (0.8)	
Unknown/Other	77 (1.9)	67 (1.7)	10 (7.8)	
**Comorbidities, no. (%)**				
Congestive heart failure	1118 (27.7)	1072 (27.4)	46 (35.9)	**0.034, 1.49 (1.03, 2.15)**
COPD	1310 (32.4)	1274 (32.6)	36 (28.1)	0.29, 0.81 (0.55, 1.20)
Diabetes	1220 (30.2)	1185 (30.3)	35 (27.3)	0.474, 0.87 (0.58, 1.29)
Chronic kidney disease	1159 (28.7)	1108 (28.3)	51 (39.8)	**0.005, 1.68 (1.17, 2.40)**
Malignancy	948 (23.5)	917 (23.4)	31 (24.2)	0.839, 1.04 (0.69, 1.57)
**Neutrophil/lymphocyte ratio, median, (IQR)**	9.26 (5.13, 16.35)	9.2 (5.08, 16.18)	11.62 (6.4, 21.66)	**0.007**
**Clinical Severity Scores**				
Pneumonia Severity Index, median (IQR)	113 (87, 141)	112 (86, 140)	154 (131, 183)	**<0.001**
CURB 65	3 (2, 3)	3 (2, 3)	4 (3, 4)	**<0.001**
APACHE III	65 (52, 80)	64 (51, 78)	82 (67, 106)	**<0.001**

APACHE: Acute Physiology and Chronic Health Evaluation, CI: Confidence interval, COPD: Chronic Obstructive Pulmonary Disease, NLR: Neutrophil Lymphocyte ratio, IQR: interquartile range, OR: Odds ratio.

* To address the issue of a high number of cells having an expected count of less than 5, the race categories were merged for comparison purposes into two groups: White and others.

* To address the issue of a high number of cells having an expected count of less than 5, the ethicity categories were merged for comparison purposes into two groups: non-Hispanic and others.
